# The effects of a physiotherapist-led exercise intervention on peripheral neuropathy among people living with HIV on antiretroviral therapy in Kigali, Rwanda

**DOI:** 10.4102/sajp.v75i1.1328

**Published:** 2019-08-12

**Authors:** David K. Tumusiime, Aimée Stewart, Francois W.D. Venter, Eustasius Musenge

**Affiliations:** 1 College of Medicine and Health Sciences, University of Rwanda, Kigali, Rwanda; 2 Department of Physiotherapy, School of Therapeutic Sciences, University of the Witwatersrand, Johannesburg, South Africa; 3 Witwatersrand Reproductive Health and HIV Institute (WRHI), Johannesburg, South Africa; 4 Department of Medicine, University of the Witwatersrand, Johannesburg, South Africa; 5 Division of Biostatistics and Epidemiology, School of Public Health, University of Witwatersrand, Johannesburg, South Africa

**Keywords:** peripheral neuropathy, physiotherapy, exercise, HIV, ART, randomised controlled trial, Rwanda

## Abstract

**Background:**

HIV-associated peripheral neuropathy (PN) is common in people living with HIV. Its management is mostly symptomatic utilising pharmacological approaches.

**Objectives:**

This study determined the effects of an exercise intervention on PN among Rwandan people living with HIV receiving antiretroviral therapy (ART).

**Methods:**

A 12-week single-blinded randomised controlled trial using the Brief Peripheral Neuropathy Screen (BPNS) as the assessment tool tested the effects of an exercise intervention on PN, followed by a 12-week non-intervention period. A total of 120 people with HIV- associated PN on ART were randomised to an exercise or no exercise group. Both groups continued receiving routine care. A bivariate analysis using Pearson’s chi-square test for significant differences in PN symptoms and signs, between groups, at baseline, after the 12 weeks intervention and 12 weeks post-intervention using generalised linear regression models to determine predictors of treatment outcomes was undertaken, utilising an intention-to-treat analysis (alpha *p* ≤ 0.05).

**Results:**

At 12 weeks, the intervention group compared to the control: neuropathic pain 70% versus 94% (*p* < 0.005), PN symptoms severity – mild and/or none in 85% versus 60% (*p* < 0.001) and radiation of PN symptoms reduced, 80% versus 37% (*p* < 0.001). There were no differences in PN signs at 12 weeks intervention and at 12 weeks post-intervention. Having changed the antiretroviral (ARV) and having developed PN symptoms after the start on ARVs predicted treatment improvement, while demographic factors did not predict any treatment outcome.

**Conclusion:**

A physiotherapist-led exercise intervention improved PN symptoms, but with non-significant improvement in PN signs. Factors related to early diagnosis and treatment of PN were facilitators for the improvement of PN symptoms.

**Clinical implications:**

Physiotherapist-led exercises should be integrated into the routine management of people living with HIV on ART with PN symptoms.

## Background

HIV-associated peripheral neuropathy (HIV-PN) is one of the most common neurological conditions in people living with HIV (PLHIV) (Ellis et al. 2010; Evans et al. 2011; Phillips et al. 2014; Wang et al. 2014). The most common PN is distal sensory polyneuropathy (DSP) often assessed and diagnosed with the Brief Peripheral Neuropathy Screen (BPNS) (Tumusiime et al. 2014a).

Both pharmacological and non-pharmacological modalities are recommended for the management of PN (Nicholas et al. 2007). A systematic review conducted by Phillips et al. (2010) to determine the effectiveness of pharmacological management of HIV-PN indicated that only tropical capsaicin 8%, smoked cannabis and recombinant human nerve growth factor (rhNGF), reduced HIV-PN more than placebo. However, the authors concluded that rhNGF is not clinically available and smoked cannabis cannot be recommended for routine treatment, thus new strategies for managing HIV-PN are required (Phillips et al. 2014).

Non-pharmacological management that includes exercise programmes and lifestyle changes is recommended (Ahmad & Goucke 2002). However, most of the non-pharmacological management benefits described are for diabetes-related PN, and these include nutritional supplements, warm water footbaths, rest, elevation of limbs, massage and exercises (Taylor et al. 2007). There is limited literature on the effects of these non-pharmacological modalities, particularly exercise for the management of HIV-PN. The available literature on exercise and PN showing beneficial results for diabetes-related PN (Adeniyi et al. 2010) describes strengthening and balance training incorporated into aerobic exercise programmes (Tofthagen, Visovsky & Berry 2012; White, Pritchard & Turner-Stokes 2004). Thus, it is hypothesised that exercise would also be effective in the prevention or reduction of PN symptoms and signs because of various causes, including HIV-PN (Dobson, McMillan & Li 2014).

The existing evidence shows that exercises are safe and affordable in PLHIV (Mutimura et al. 2008; Nixon et al. 2005; O’Brien et al. 2010). The systematic review by Nixon et al. (2005) examined the safety and effectiveness of exercise among PLHIV. They indicated that ‘exercise appears to be safe and may lead to significant reductions in depressive symptoms and potentially clinically important improvements in cardiopulmonary fitness’ (Nixon et al. 2005:2). Similar findings were identified in a systematic review by O’Brien et al. (2010). Mutimura et al. (2008) found that an exercise intervention improves components of quality of life (QoL) of PLHIV (Mutimura et al. 2008).

There is a paucity of reported effects of aerobic exercise interventions specifically for PLHIV with PN. Hence, it became important to test the effects of a physiotherapist-led exercise (PTExs) intervention in a randomised controlled trial (RCT). Thus, the purpose of this study was to determine if an exercise intervention has an effect on PN among PLHIV on ART in Rwanda, given that exercise is a simple inexpensive intervention that could potentially be rolled out to PLHIV to improve PN.

## Methods

### Study design, site, participants and selection

This RCT included participants who attended four randomly selected ART clinics situated in health centres and hospitals in Kigali, Rwanda. The exercise intervention was provided to the participants at the Kigali Health Institute’s physiotherapy clinic and fitness centre. Included in the study were male and female PLHIV on ART aged 18 and above; thus, the participants in the study were people already diagnosed with HIV at the HIV Voluntary Counselling and Test programmes at health centres and hospitals in Rwanda. The study recruited those with PN and on ART from the ART clinics.

According to the American College of Sports Medicine guidelines for exercise prescription for PLHIV (Sonya 2006), participants were excluded if they had active opportunistic infections, a clinical history contraindicating exercise (Ortiz 2014), could not walk without support, had musculoskeletal and central neurological impairments, a known history of diabetes or substance abuse, vitamin B12 deficiency, tuberculosis, renal failure, hypothyroidism and any other pathologies associated with neuropathy.

The sample considered all 164 PLHIV who had been randomly screened in a prevalence study on PN prior to this trial, by the same authors (Tumusiime et al. 2014). Hence, potential participants were consecutively invited from the 164 PLHIV with PN on ART. Out of the 164 participants, 139 responded positively to the invitation: 19 were excluded based on the above-mentioned exclusion criteria for this study and 120 were then eligible to participate. Sample size computation was done to check if the 120 had sufficient power to detect a difference in PN between the two groups (effect size; *p* = 0.05). The calculation was done by allowing for a dropout of a maximum of 30% per group based on previous similar studies such as that of Mutimura et al. (2008). With this consideration, the sample size of 120, randomised to 60 per group, had 80% power to detect the effect.

A total of 120 participants were assessed at baseline and were then block randomised into 60 in the experimental and 60 in the control group, using computer-generated random numbers. Concealed allocation to groups was assured by an independent research assistant placing the numbers into opaque sealed envelopes which were then given to eligible participants by a second research assistant. The study was single blinded, the assessor (first author) being blinded to group allocation, until after the last assessments.

The experimental group continued receiving Routine Health Care (RHC), antiretrovirals (ARVs) and other prophylactic medications, namely analgesics, antidepressants, multivitamins, as well as routine medical consultations, CD4 testing and counselling services, from the respective ART clinics plus physiotherapy-led exercises (RHC + PTExs). The control group received only RHC.

The PTExs comprised an exercise intervention that was identified from the literature (Mutimura et al. 2008; O’Brien et al. 2010; White et al. 2004) and was recommended as safe and beneficial to PLHIV. The PTExs consisting of aerobic group exercises were given to the experimental group in a well-organised gymnasium. The group included both female and male participants. The intervention included a 15-min warm-up of walking (slow to brisk walk plus full range of upper and lower limb flexibility exercises); 15 min of mobility training with self-stretching (mainly dynamic stretching) in standing, lying and long sitting; 10 min of muscle conditioning with isometric exercises in various starting positions; 10 min of balance exercises and finally 10 min of cooling down (stretching and deep breathing).

Participants’ blood pressures and respiratory rates were carefully monitored before and after the exercise sessions based on standard procedures as used in a similar study (Mutimura et al. 2008). Each session was 60 min in duration, offered 3 times a week for 12 weeks. In addition, participants were educated on how to exercise on their own at home. This was so that after the supervised exercise sessions of 12 weeks, participants would continue exercising on their own at home. They were assessed at baseline, after the 12 weeks of the intervention and then again after a further 12 weeks. A qualified physiotherapist, trained by the first author, conducted the exercise programme. Participants were reminded about the programme telephonically at regular intervals, and monthly calls were made to the control group by a designated research assistant after the baseline assessments to remind them of the assessments at 12 and 24 weeks after the start of the study. Assessments were done by the blinded first author. The BPNS which had been adapted in a study previous to this one (Tumusiime et al. 2014) and a checklist for demographic and health status characteristics were used to evaluate the PN.

### Measures

The BPNS assessed the primary outcome of interest, namely the PN. The Brief Peripheral Neuropathy Screen is both a self-report and objective PN screening tool, which is a valid and reliable instrument for diagnosis of PN, specifically DSP (Cherry et al. 2005), with a specificity of 88% and sensitivity of 78% (Cherry et al. 2005). The BPNS examines subjective and objective outcomes consistent with PN. It has been used in several clinical trial protocols, in particular by the AIDS Clinical Trial Group (ACTG) (Evans et al. 2011), and evaluated in large-scale studies. It was identified to precisely discover PLHIV who have the highest grade of peripheral nervous system dysfunction and pathology.

Cherry et al. (2005) concluded that the presence of both symptoms and signs on the BPNS provides a useful operational criterion for HIV- PN in the era of ART. One of the studies by Mehta et al. (2011) implemented the validated BPNS in patients receiving ART in Mombasa, Kenya, and they recommended that the tool can be easily integrated into routine care by general practitioners in an outpatient HIV clinic with limited resources. The tool was, therefore, realistic to use in a resource-limited country like Rwanda because of the high cost and lack of specialised instruments for nerve conduction, such as electromyography or sural nerve testing and electrophysiological changes. However, the tool was piloted for adaptation to assess PN among PLHIV on ART in Rwanda (Tumusiime et al. 2014a).

As described in the above study (Tumusiime et al. 2014a), BPNS is both a self-report and objective PN screening tool. It is both a valid and reliable instrument for diagnosis of PN specifically DSP, where ‘PN is defined as the combination of at least one subjective neuropathy grade greater than 0 and either reduced or no sense of vibration and/or reduced/no ankle deep tendon reflex, bilaterally’ (Tumusiime et al. 2014b). At both baseline and post-intervention, the participants were requested to score the ‘presence and severity of symptoms, using a scale of 1 (mild) to 10 (severe) for each leg separately’. The symptoms assessed were as follows: ‘pain, aching, or burning in feet and/or legs; pins and needles in feet and/or legs; and numbness in feet and/or legs’.

The single highest score of the six scores (three for each leg) was transformed to a:

[*S*]ubjective PN grade as follows: symptoms absent = grade 0, score of 1–3 = grade 1, score of 4–6 = grade 2, and score of 7–10 = grade 3. (Mehta et al. 2011:491)

Symptoms had to be bilateral to be graded as ≥ 1. ‘Objective findings (signs) included in the BPNS are the loss of the sense of vibration and abnormal ankle deep tendon reflexes’. The vibration sense perception was assessed with a 128-Hz tuning fork, ‘maximally struck and applied at the great toe distal interphalangeal joint of each foot’. The vibration sense was defined as ‘normal for a vibration felt for > 10 s, mild loss for a vibration felt for 6–10 s, moderate loss for a vibration felt for ≥ 5 s, and severe loss for no feeling of vibration’. The ankle tendon reflexes were defined as ‘absent, hypoactive, normal, hyperactive, or clonus’.

For testing of the ankle reflex, the participant was in a sitting position and the assessor (first author) used one hand to place the participant’s foot into dorsiflexion at 90°. Holding the reflex hammer in the other hand, the assessor struck the ankle tendon. The tendon reflex was felt in the assessor’s hand as planter flexion of the participant’s foot.

Improvement was determined as the differences in positive change between the two groups, that is the reduced percentages of people with PN, which was considered as an improvement in PN.

A demographic and health status questionnaire was utilised to obtain basic data from the participants.

### Data analysis

This included tabulations for frequencies and percentages for categorical variables and measures of central tendency (means) and measures of variability (standard deviations) for continuous variables. A bivariate analysis using Pearson’s chi-square test for significant differences between the experimental and control groups, at baseline, after 12 weeks of PTExs and at 12 weeks post-intervention, was done. Because we were interested in overall group changes over time, we adopted generalised estimating equations (GEE) from the generalised linear models with a logit canonical link function to identify the significant predictors of treatment outcome. The predictors tested included the demographic and health status-related characteristics: age, gender, level of education, marital status, occupation, duration since HIV diagnosis, CD4 cell count levels, duration on ART, treatment regime (ARVs), ARV regimen changes and whether PN started before or after the start on ARVs. Adjusted odds ratios and their 95% confidence interval (95% CI) values were reported on magnitude of association and prediction on PN as binomial outcomes. Significance level acceptance was set at *p* < 0.05.

Data were analysed using STATA (version 11; STATA Corp, College Station, TX, USA). All analyses were done as intention to treat, which maintained the original group composition achieved by randomisation.

### Ethical considerations

An ethical clearance certificate (protocol number M080812) was obtained from the Human Research Ethics Committee of the University of the Witwatersrand. As the data were collected in Rwanda, national ethical clearance (no. 032IRB052011) was also obtained from the Institutional Review Board at Kigali Health Institute and the proposal was scientifically approved by the National Commission for Control of HIV and AIDS (approval letter no. 0137/CNLS/2011/S.E) in Rwanda. Participants gave written signed informed consent prior to their participation in the study and gave permission to use their medical records or files. This study was retrospectively registered with the Pan African Clinical Trial Registry (PACTR201803003147805).

## Results

**FIGURE 1:**
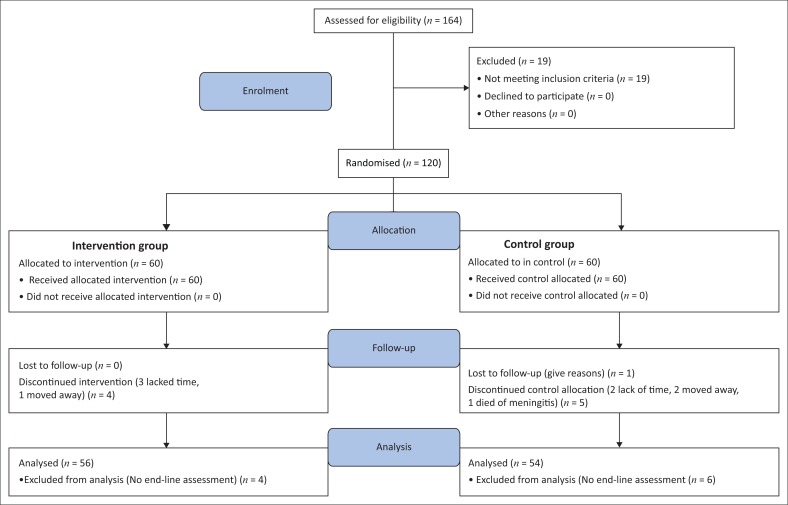
Flow diagram.

Out of 120 participants who were randomised into the intervention and control groups, 93% participated in all exercise sessions (Figure 1).

There were no significant differences in the demographic and health status characteristics between the experimental and control groups at baseline (Table 1).

**TABLE 1:** Demographic and health status characteristics of the participants: comparison between the experimental and control groups at baseline (*n* = 120).

Characteristics	Experimental group (*n* = 60)	Control group(*n* = 60)	*p*
Age	41.2 ± 7.8 (mean ± s.d. years)	40.4 ± 7.7 (mean ± s.d. years)	0.59
**Gender**			
Female	48 (80)	50 (83)	0.64
Male	12 (20)	10 (17)	
**Education**			
No schooling	11 (18)	13 (22)	0.54
Some primary school	35 (58)	29 (48 )	
Some secondary and university education	14 (24)	18 (30)	
**Occupation**			
Employed	5 (8)	6 (10)	0.51
Self-employed/peasant/farmers	13 (21)	18 (30)	
Unemployed	4 (70)	36 (60)	
**Marital status**			
Single	1 (2)	2 (3)	0.10
Married	16 (26)	18 (30)	
Separated/divorced	4 (7)	12 (20)	
Widow/widower	39 (65)	28 (48)	
**Duration since HIV diagnosis**			
Less or equal to 3 years ago	8(13)	3 (5)	0.31
4–6 years ago	18 (30)	19 32)	
7 and above years ago	34 (57)	38 (63)	
**CD4 cell count**			
≤ 350	22 (37)	15 (25)	0.17
> 351	38 (63)	45 (75)	
**Duration on ARVs**			
Less or equal to 3 years ago	15 (25)	18 (30)	0.82
4–6 years ago	36 (60)	34 (54)	
7 and above years ago	9 (15)	8 (13)	
**ARV regimen combination started with**			
None D4T including	33 (55)	40(66)	0.78
D4T including	27 (45)	20 (34)	
**Current ARV regimens’ combination**			
None D4T including	53 (88)	50 (83)	0.60
D4T including	7 (12)	10 (17)	
**ARV regimen changes since started on ART**			
No change	18 (30)	16 (27)	0.69
One or more changes	42 (70)	44 (73)	
**The onset of PN symptoms and signs**			
Before starting on ARVs	**5 (8)**	12 (20)	0.07
After starting on ARVs	**55 (92)**	48 (80)	
**After how long on ARVs when PNS started**			
Within the first 12 months	27 (45)	23 (39)	0.09
After the first 12 months	33 (55)	37 (61)	

Note: Data are expressed as number (%) unless otherwise specified.

ARV, antiretroviral; CD4, cluster differentiation 4; PN, peripheral neuropathy; PNS, Peripheral Neuropathy Screen; s.d., standard deviation.

**FIGURE 2:**
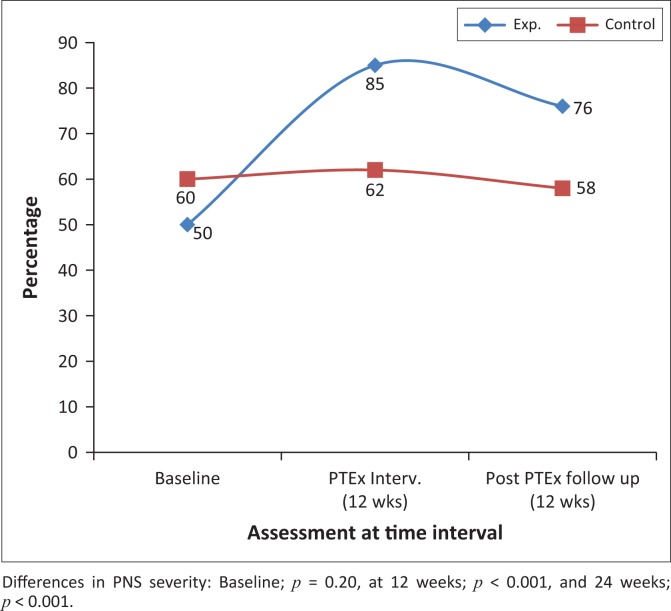
The percentage of participants with mild to no peripheral neuropathy symptoms at baseline, 12 and 24 weeks, in the intervention and control groups.

**FIGURE 3:**
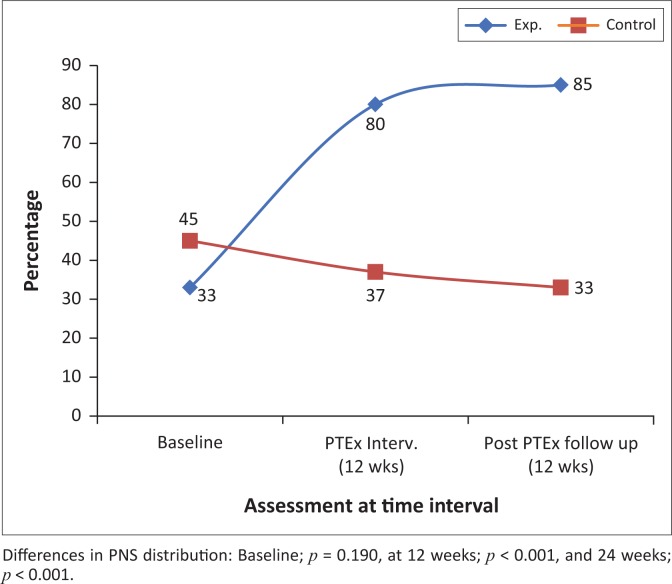
The percentage of participants with peripheral neuropathy symptoms distribution to various parts of the lower extremity, at baseline, 12 and 24 weeks, in the intervention and control groups.

**FIGURE 4:**
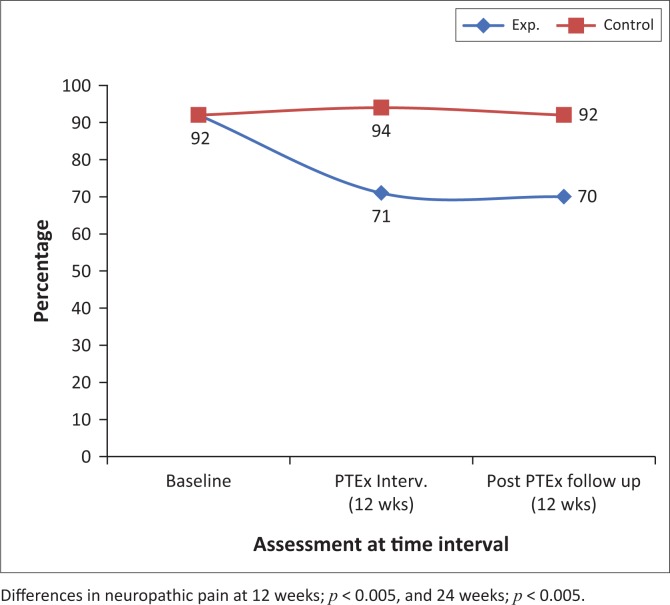
The percentages of participants with neuropathic pain at baseline, 12 and 24 weeks, in the intervention and control groups.

Figures 2–4 and 7 demonstrate the increased percentage of participants whose symptoms significantly improved in the exercise intervention group as compared to the control group both after the intervention and again after a further 12 weeks.

**FIGURE 5:**
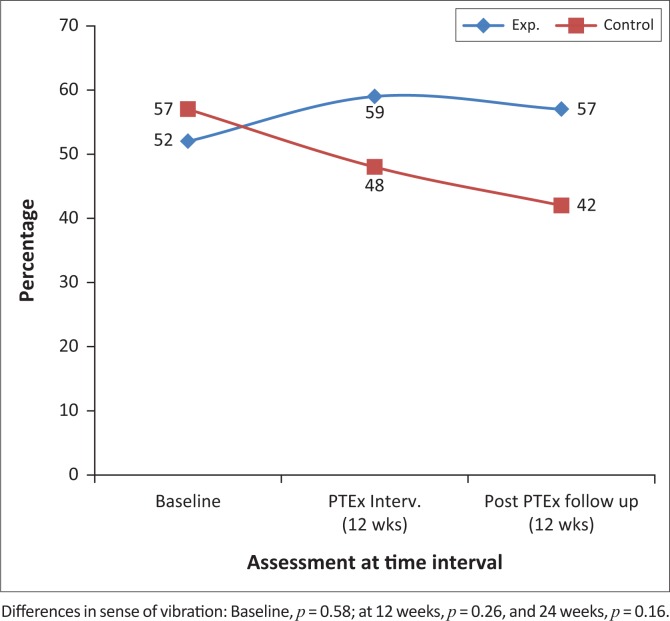
The percentages of participants with normal or minimally reduced vibration sense at baseline, 12 and 24 weeks, in the intervention and control groups.

**FIGURE 6:**
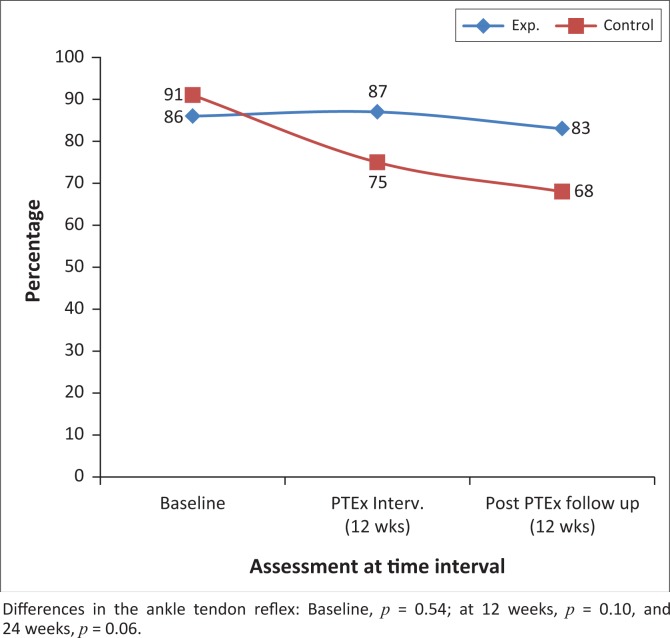
The percentages of participants with normal or minimally ankle tendon reflex at baseline, 12 and 24 weeks, in the intervention and control groups.

There were no significant differences in the signs of PN between the intervention and control groups at the end of the 12 weeks of intervention and again after a further 12 weeks, as can be seen in Figures 5 and 6.

**FIGURE 7:**
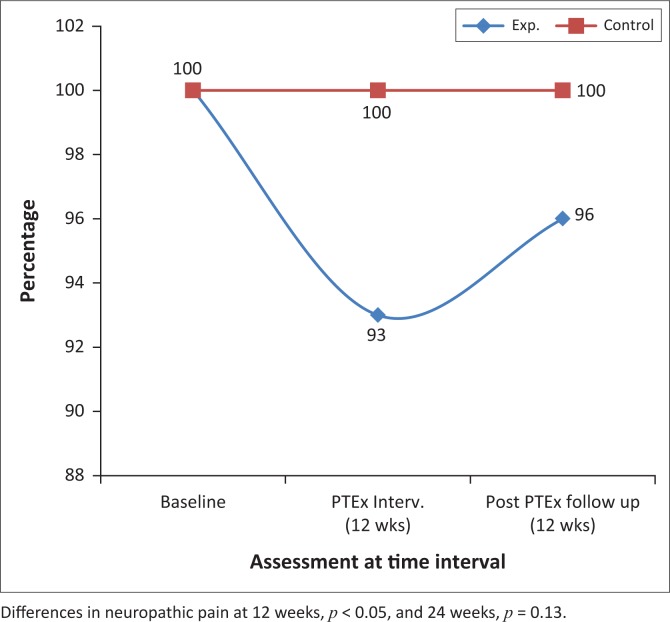
The percentages of participants with peripheral neuropathy at baseline, 12 and 24 weeks, in the intervention and control groups.

The results of the univariate and multivariate analyses determined the predictors for treatment outcome (Table 2). No demographic factors were associated with the treatment outcome. Statistically significant health-related characteristics in the univariate model reporting odds ratio (OR) were further confirmed with multivariate analysis models with adjusted odds ratios (aORs). The participants who were diagnosed with HIV for 7 years and longer were likely to have less improvement (OR = 0.4, 95% CI [0.2–1.0]; *p* = 0.05 and aOR = 0.4, 95% CI [0.2–0.9]; *p* = 0.02) compared to 3 years or less since the diagnosis. Having changed the ARV regimen was likely to predict improvement (OR = 1.6, 95% CI [1.0–2.7]; *p* = 0.05 and aOR = 1.7, 95% CI [1.0–3.0] *p* = 0.04) compared to no change of ARV regimen. The participants who developed PN symptoms after the start on ARVs were likely to improve (OR = 2.1, 95% CI [1.1–3.8]; *p* = 0.01 and aOR = 2.2, 95% CI [1.2–4.2]; *p* < 0.01).

**TABLE 2:** Generalised linear regression with general estimating equation for the associated and influencing factors of improvement on peripheral neuropathy.

Factors	Univariate analysis		Multivariate analysis	
	Univariable (OR) (95% CI)	*p*	Adjusted odds ratio (aOR) (95% CI)	*p*
Age in years (mean ± s.d.)	1.0 (1.0–1.1)	0.12	-	-
**Gender**				
Male	0.9 (0.5–1.7)	0.75	-	-
Female	-	-	-	-
**Marital status**				
Unmarried	1.0 (ref.)	-	-	-
Married	1.2 (0.3–5.1)	0.77	-	-
Separated/divorced/widowed	1.6 (0.4–5.9)	0.51	-	-
**Occupation**				
Employed	1.0 (ref.)	-	-	-
Self-employed/peasant	1.5 (0.6–3.7)	0.35	-	-
Unemployed	1.7 (0.8–4.0)	0.20	-	-
**Level of education**				
No schooling	1.0 (ref.)		-	-
Primary	0.9 (0.5–1.7)	0.73	-	-
≥ Secondary	0.6 (0.3–1.2)	0.15	-	-
**Duration since HIV diagnosis**				
Less than or equal to 3 years	1.0 (ref.)	-	-	-
4–6 years	0.5 (0.2–1.2)	0.12	0.4 (0.2–1.0)	0.06
7 and above years	0.4 (0.2–1.0)	0.05	0.4 (0.2–0.9)	0.02*
**CD4 cell count**				
≤ 350	-	-	-	-
> 351	1.4 (0.8–2.3)	0.24	-	-
**Duration on ARV**				
Less than or equal to 3 years	1.0 (ref.)	-	-	-
4–6 years	0.8 (0.5–1.3)	0.39	-	-
7 and above years	1.1 (0.5–2.1)	0.89	-	-
**ARV regimen combination started with**				
Non-D4T containing	-	-	-	-
D4T containing	0.7 (0.4–1.2)	0.14	-	-
**Current ARV regimens’ combination**				
Non-D4T containing	-	-	-	-
D4T containing	0.6 (0.3–1.3)	0.21	-	-
**ARV regimen changes since started on ART**				
No change				
One or more changes	1.6 (1.0–2.7)	0.05	1.7 (1.0–3.0)	0.04*
**The onset of PN symptoms and signs**				
Before starting on ARVs	-	-	-	-
After starting on ARVs	2.1 (1.1–3.8)	0.01	2.2 (1.2–4.2)	< 0.01*

ARV, antiretroviral; CD4, cluster differentiation 4; PN, peripheral neuropathy; s.d., standard deviation.

* , Statistically significant for the factors associated and influencing improvement of PN.

## Discussion

The exercise intervention reduced PN symptoms among PLHIV on ART who exercised for 12 weeks, and this reduction was sustained for a further 12 weeks. Therapeutic exercises have known beneficial effects on painful neuromuscular conditions, reduce functional activity limitations and improve QoL through enhancing participation in activities of daily living (ADL) (Cherry et al. 2005), but there is insufficient existing information on the effects of exercise on PN symptoms in PLHIV on ART particularly in resource-limited settings (Taylor et al. 2007).

Exercise is considered to be a key strategy by rehabilitation professionals to address the disabling health-related consequences of HIV (Stewart et al. 2008). Several studies with exercise interventions on PLHIV have been conducted, including studies identified in systematic reviews (Maharaj & Chetty 2011; Taylor et al. 2007; Nixon et al. 2005). However, in all of these studies there is a dearth of evidence on the effects of exercise on PN among PLHIV on ART. To our knowledge, there is only one study that has been reported and that is a pilot study, without a control group and it has inconclusive results (Sandoval, Runft & Roddey 2010).

In our study, there were reductions in the neuropathic pain, numbness, paraesthesia and distribution of the symptoms along the lower extremities. To our knowledge, this study is the first one to show that a physiotherapist-led exercise intervention can reduce PN symptoms in PLHIV on ART in Rwanda. The findings are similar to the few existing studies, particularly the study by Sandoval et al. (2010) who found that a community exercise intervention reduced activity limitations among PLHIV with inflammatory peripheral neuropathy; however, their study was not a RCT.

The mechanism of how exercise reduces symptoms of PN is not yet clear. Exercise has been known to have beneficial effects such as an antidepressant effect, reduction of anxiety and inducement of sleep for those with insomnia, among others (Schuch et al. 2014; Suna et al. 2015). The antidepressant effect (Schuch et al. 2014; Suna et al. 2015) might play a role and is one of the treatments of choice for neuropathic pain in PLHIV (Eng & Reime 2014; Lana, Lérida & Mendoza 2008). The antidepressant effect of exercise is likely a result of the increased blood circulation that enhances the discharge of neuro-chemical pain inhibitors (Baron, Binder & Wasner 2010; Brix Finnerup, Hein Sindrup & Staehelin Jensen 2013). These discharged pain inhibitors inhibit pain receptors by enhancing the closing of the pain gate at the spinal dorsal horn of the small diameter nerves (Dobson et al. 2014), thus relieving pain. In addition, exercise leads to a feeling of relaxation, hence improving mood. In cases of pain, the feeling of good mood as a result of exercise inhibits the nociceptive mechanism and releases neuro-chemical pain inhibitors, hence modifying pain perception (Dworkin et al. 2003).

The exercises were prescribed and supervised by a physiotherapist, and the usual therapeutic exercise techniques were applied. These included stretching for joint mobility, isometric strengthening, and aerobic exercises for ‘warm-up’ and finally ‘cool down’ at the end of every session. Stretching exercises after warming up are known for their effect on improving flexibility (Golan 2008; Yaksh & Sorkin 2005) and enhancing pain-free range of movements about a joint to promote better performance (Hess & Woollacott 2005; Zakas et al. 2006). Neuromuscular flexibility has been reported as a benefit of stretching before performing strength training exercises (Marek et al., 2005; Rubini, Costa & Gomes 2007; Sigal et al. 2004).

The stretching exercises might have induced muscle relaxation, consequently improving pain-free movements (Rubini et al. 2007). For example, most of the participants anecdotally reported being able to squat on a pit latrine, and walk up and down stairs more easily after the intervention while they had expressed difficulty with these activities before the intervention. Therefore, with the participants able to achieve pain-free movements, they reported reduced PN symptoms.

Furthermore, stretching exercises are reported to improve neural tissue flexibility. The stretching mechanism includes ‘sliding and tensioning techniques, which are thought to enhance nerve gliding and restore neural tissue mobility’ (Dobson et al. 2014). This may have induced nerve flexibility and mobility (Oskay et al. 2010), resulting in reduced pain during activities. The reduction of pain was of considerable benefit to these participants.

To our knowledge, the only study that has tested the effects of exercise on neuropathic pain in PLHIV is a pilot study by Sandoval et al. (Dobson et al. 2014), with few participants (Sandoval et al. 2010), and no control group, and it did not show changes in pain. In our study, the effect of PTExs on neuropathic pain included reduced sensations such as burning, pins and needles and tingling sensations, numbness, painful cold, pains in the form of ‘electric shocks’ hyper and hypo-aesthesia to touch and pin prick.

However, there were no significant improvements found in the PN signs, namely sense of vibration and ankle tendon reflex. Sense of vibration and tendon reflexes are mediated by large sensory nerves, unlike PN symptoms which occur as a result of small sensory nerve fibre damage (O’Brien et al. 2008). In addition, the development of PN signs is progressive and takes some time, though the time frame is uncertain. Consequently, it is possible that the treatment and relief or recovery of PN signs may occur after a longer duration exercise intervention than the one conducted here. The improvements shown in the PN symptoms may be because the recovery is quicker in small nerve fibres than in large fibres. Thus, the change in the improvement of the PN signs may need to be evaluated after a longer period of intervention. It seems that the improvement of the PN symptoms was as a result of the improvement in the neuro-tissue flexibility and in mobility, and possibly an improvement in the nerve regeneration or growth.

The improvement attained by the participants during the 12 weeks of exercise was maintained 12 weeks post-exercise, although there was a slight decline in effect. This is likely attributable to the probable continuation of exercises by the participants after the structured exercise programme, though this was not assessed. Further studies could evaluate a home exercise programme among PLHIV with PN. This could encourage PLHIV to continue exercising as a lifetime strategy to prevent and reduce the impact of PN (Devigili et al. 2008).

Some factors significantly predicted treatment outcome. The predictors for improvement of PN with exercises included health-related characteristics, namely the duration of HIV infection, ART regimen changes and PN that developed post-ART initiation. The participants diagnosed with HIV for 7 years and longer were likely to have less improvement as a result of the exercise intervention. Because HIV infection might be one of the causes of PN (Dobson et al. 2014), this implies that living with the infection for a long time results in more severe PN or that prolonged exposure to HIV may render the nerve damage permanent. Early screening of the PN immediately after a person is diagnosed, and treating appropriately is important. Peripheral neuropathy is a chronic and disabling condition and early management that includes physiotherapist-led exercises might give relief and better health outcomes.

The participants who developed PN after initiating ART were likely to significantly improve compared to those who developed PN before initiating ART. The PN that develops after initiating ART is more likely to be related to ART than HIV. A change in the drug regimen (if the regimen contains medication associated with PN such as stavudine or tenofovir) may facilitate improvement of the pain, and with exercise as a supplementary treatment the improvement may be quicker. It is, therefore, important to include exercise in addition to drug regimen changes in the management of PN that occurs as a result of ART.

The participants in whom the ARV regimen was not changed were also likely to improve. This is possible because it is either that the PN was not severe, the PN had just started or it was not suspected to be augmented with a particular toxic ARV type (Dubey et al. 2013).

## Conclusions and recommendations

Physiotherapist-led exercise seems to improve PN symptoms as the results of this study indicate that all the PN symptoms among the participants who exercised for 12 weeks improved as compared to the control group which had no improvement. Importantly, this improvement was maintained for a further 12 weeks post-intervention. This is likely a result of the probable continuation of exercise by the participants after the structured exercise programme. Factors related to the early diagnosis and treatment of PN were predictors for improvements of PN.

However, there is a need for further studies to establish the mechanism of how exercise improves PN symptoms. This may lead to a better understanding of how important exercises are in the management of PN among PLHIV.

In addition, studies on the physiological effects of exercise on the toxicity of the ARVs, particularly at the mitochondrial level, may be useful.

Finally, there is a need to investigate and test exercise interventions over prolonged periods of time, to establish if there is an effect on nerve recovery, in particular the larger nerve fibres for the effect on PN signs.

## References

[CIT0001] Adeniyi A.F., Fasanmade A.A., Sanya A.O. & Borodo M., 2010, ‘Neuromusculoskeletal disorders in patients with type 2 diabetes mellitus: Outcome of a twelve-week therapeutic exercise programme’, *Nigerian Journal of Clinical Practice* 13(4), 403–408. 21220854

[CIT0002] Ahmad M. & Goucke C.R., 2002, ‘Management strategies for the treatment of neuropathic pain in the elderly’, *Drugs & Aging* 19(12), 929–945. 10.2165/00002512-200219120-00004 12495368

[CIT0003] Baron R., Binder A. & Wasner G., 2010, ‘Neuropathic pain: Diagnosis, pathophysiological mechanisms, and treatment’, *The Lancet. Neurology* 9(8), 807–819. 10.1016/S1474-4422(10)70143-5 20650402

[CIT0004] Brix Finnerup N., Hein Sindrup S. & Staehelin Jensen T., 2013, ‘Management of painful neuropathies’, *Handbook of Clinical Neurology* 115, 279–290. 10.1016/B978-0-444-52902-2.00017-5 23931787

[CIT0005] Cherry C.L., Wesselingh S.L., Lal L. & McArthur J.C., 2005, ‘Evaluation of a clinical screening tool for HIV-associated sensory neuropathies’, *Neurology* 65(11), 1778–1781. 10.1212/01.wnl.0000187119.33075.41 16344522

[CIT0006] Devigili G., Tugnoli V., Penza P., Camozzi F., Lombardi R., Melli G. et al., 2008, ‘The diagnostic criteria for small fibre neuropathy: From symptoms to neuropathology’, *Brain* 131(7), 1912–1925. 10.1093/brain/awn093 18524793 PMC2442424

[CIT0007] Dobson J.L., McMillan J. & Li L., 2014, ‘Benefits of exercise intervention in reducing neuropathic pain’, *Frontiers in Cellular Neuroscience* 8(102), 1–9. 10.3389/fncel.2014.00102 24772065 PMC3983517

[CIT0008] Dubey T.N., Raghuvanshi S.S., Sharma H. & Saxena R., 2013, ‘HIV neuropathy in pre-HAART patients and it’s correlation with risk factors in Central India’, *Neurology India* 61(5), 478–480. 10.4103/0028-3886.121912 24262448

[CIT0009] Dworkin R.H., Backonja M., Rowbotham M.C., Allen R.R., Argoff C.R. & Bennett G., 2003, ‘Advances in neuropathic pain: Diagnosis, mechanisms, and treatment recommendations’, *Archives of Neurology* 60(11), 1524–1534. 10.1001/archneur.60.11.1524 14623723

[CIT0010] Ellis R.J., Rosario D., Clifford D.B., McArthur J.C., Simpson D., Alexander T. et al., 2010, ‘Continued high prevalence and adverse clinical impact of human immunodeficiency virus-associated sensory neuropathy in the era of combination antiretroviral therapy: The CHARTER study’, *Archives of Neurology* 67(5), 552–558. 10.1001/archneurol.2010.76 20457954 PMC3924778

[CIT0011] Eng J.J. & Reime B., 2014, ‘Exercise for depressive symptoms in stroke patients: A systematic review and meta-analysis’, *Clinical Rehabilitation* 28(8), 731–739. 10.1177/0269215514523631 24535729 PMC4591069

[CIT0012] Evans S.R., Ellis R.J., Chen H., Yeh T., Lee A.J., Schifitto G. et al., 2011, ‘Peripheral neuropathy in HIV: Prevalence and risk factors’, *AIDS (London, England)* 25(7), 919–928. 10.1097/QAD.0b013e328345889d PMC3196556 21330902

[CIT0013] Golan D.E., 2008, *Principles of pharmacology: The pathophysiologic basis of drug therapy*, Lippincott Williams & Wilkins, viewed 29 July 2012, from https://books.google.rw/books?id=az8uSDkB0mgC.

[CIT0014] Hess J.A. & Woollacott M., 2005, ‘Effect of high-intensity strength-training on functional measures of balance ability in balance-impaired older adults’, *Journal of Manipulative & Physiological Therapeutics* 28(8), 582–590. 10.1016/j.jmpt.2005.08.013 16226626

[CIT0015] Lana R., Lérida A.I. & Mendoza J.L., 2008, ‘[Treatment of neuropathic pain in HIV-infected patients]’, *Enfermedades Infecciosas Y Microbiologia Clinica* 26(6), 348–355, viewed 15 June 2012, from http://www.ncbi.nlm.nih.gov/pubmed/18588818. 18588818 10.1157/13123841

[CIT0016] Maharaj S.S. & Chetty V., 2011, ‘Rehabilitation program for the quality of life for individuals on highly active antiretroviral therapy in KwaZulu-Natal, South Africa: A short report’, *International Journal of Rehabilitation Research* 34(4), 360–365. 10.1097/MRR.0b013e32834d2bab 22002109

[CIT0017] Marek S.M., Cramer J.T., Fincher A.L., Massey L.L., Dangelmaier S.M., Purkayastha S. et al., 2005, ‘Acute effects of static and proprioceptive neuromuscular facilitation stretching on muscle strength and power output’, *Journal of Athletic Training* 40(2), 94–103, viewed 09 July 2011, from http://www.ncbi.nlm.nih.gov/pubmed/15970955. 15970955 PMC1150232

[CIT0018] Mehta S.A., Ahmed A., Laverty M., Holzman R.S., Valentine F. & Sivapalasingam S., 2011, ‘Sex differences in the incidence of peripheral neuropathy among kenyans initiating antiretroviral therapy’, *Clinical Infectious Diseases* 53(5), 490–496. 10.1093/cid/cir432 21844033 PMC3156141

[CIT0019] Mutimura E., Stewart A., Crowther N.J., Yarasheski K.E. & Cade W.T., 2008, ‘The effects of exercise training on quality of life in HAART-treated HIV-positive Rwandan subjects with body fat redistribution’, *Quality of Life Research: An International Journal of Quality of Life Aspects of Treatment, Care and Rehabilitation* 17(3), 377–385. 10.1007/s11136-008-9319-4 PMC3167195 18320351

[CIT0020] Nicholas P.K., Mauceri L., Slate Ciampa A., Corless I.B., Raymond N., Barry D.J. et al., 2007, ‘Distal sensory polyneuropathy in the context of HIV/AIDS’, *The Journal of the Association of Nurses in AIDS Care: JANAC* 18(4), 32–40. 10.1016/j.jana.2007.05.003 17662922

[CIT0021] Nixon S., O’Brien K., Glazier R.H. & Tynan A.M., 2005, ‘Aerobic exercise interventions for adults living with HIV/AIDS’, *The Cochrane Database of Systematic Reviews* 2, CD001796 10.1002/14651858.CD001796.pub2 15846623

[CIT0022] O’Brien K., Tynan A.-M., Nixon S. & Glazier R.H., 2008, ‘Effects of progressive resistive exercise in adults living with HIV/AIDS: Systematic review and meta-analysis of randomized trials’, *AIDS Care* 20(6), 631–653. 10.1080/09540120701661708 18576165

[CIT0023] O’Brien K., Nixon S., Tynan A.-M. & Glazier R., 2010, ‘Aerobic exercise interventions for adults living with HIV/AIDS’, *The Cochrane Database of Systematic Reviews* 8, CD001796 10.1002/14651858.CD001796.pub3 PMC7061352 20687068

[CIT0024] Ortiz A., 2014, ‘Exercise for adults living with human immunodeficiency virus infection in the era of highly active antiretroviral therapy’, *International Journal of Physical Medicine & Rehabilitation* 2(4), 1–4. 10.4172/2329-9096.1000213

[CIT0025] Oskay D., Meriç A., Kirdi N., Firat T., Ayhan C. & Leblebicioğlu G., 2010, ‘Neurodynamic mobilization in the conservative treatment of cubital tunnel syndrome: Long-term follow-up of 7 cases’, *Journal of Manipulative and Physiological Therapeutics* 33(2), 156–163. 10.1016/j.jmpt.2009.12.001 20170781

[CIT0026] Phillips T.J.C., Brown M., Ramirez J.D., Perkins J., Woldeamanuel Y.W. & Williams A., 2010, ‘Pharmacological treatment of painful HIV-associated sensory neuropathy: A systematic review and meta-analysis of randomised controlled trials’, *PLoS One* 5(12), e14433 10.1371/journal.pone.0014433 21203440 PMC3010990

[CIT0027] Phillips T.J.C., Brown M., Ramirez J.D., Perkins J., Woldeamanuel Y.W., Williams A.C. et al., 2014, ‘Sensory, psychological, and metabolic dysfunction in HIV-associated peripheral neuropathy: A cross-sectional deep profiling study’, *Pain* 155(9), 1846–1860. 10.1016/j.pain.2014.06.014 24973717 PMC4165602

[CIT0028] Rubini E.C., Costa A.L.L. & Gomes P.S.C., 2007, ‘The effects of stretching on strength performance’, *Sports Medicine (Auckland, N.Z.)* 37(3), 213–224, viewed 20 April 2012, from http://www.ncbi.nlm.nih.gov/pubmed/17326697. 10.2165/00007256-200737030-00003 17326697

[CIT0029] Sandoval R., Runft B. & Roddey T., 2010, ‘Pilot study: Does lower extremity night splinting assist in the management of painful peripheral neuropathy in the HIV/AIDS population?’, *Journal of the International Association of Physicians in AIDS Care (Chicago, Ill.: 2002)* 9(6), 368–381. 10.1177/1545109710373828 21075912

[CIT0030] Schuch F.B., Pinto S.S., Bagatini N.C., Zaffari P., Alberton C.L., Cadore E.L. et al., 2014, ‘Water-based exercise and quality of life in women: The role of depressive symptoms’, *Women & Health* 54(2), 161–175. 10.1080/03630242.2013.870634 24329155

[CIT0031] Sigal R.J., Kenny G.P., Wasserman D.H. & Castaneda-Sceppa C., 2004, ‘Physical activity/exercise and type 2 diabetes’, *Diabetes Care* 27(10), 2518–2539. 10.2337/diacare.27.10.2518 15451933

[CIT0032] Sonya L.A., 2006, ‘Physical therapy for patients with HIVAIDS’, *Cardiopulmonary Physical Therapy Journal* 17(3), 103–109. 10.1097/01823246-200617030-00002

[CIT0033] Stewart A., Cade T.W., Mutimura E., Yarasheski K.E. & Crowther N.J., 2008, ‘Exercise training reduces central adiposity and improves metabolic indices in HAART-treated HIV-positive subjects in Rwanda: A randomized controlled trial’, *AIDS Research and Human Retroviruses* 24(1), 15–23. 10.1089/aid.2007.0023 18275343 PMC3936606

[CIT0034] Suna J.M., Mudge A., Stewart I., Marquart L., O’Rourke P. & Scott A., 2015, ‘The effect of a supervised exercise training programme on sleep quality in recently discharged heart failure patients’, *European Journal of Cardiovascular Nursing: Journal of the Working Group on Cardiovascular Nursing of the European Society of Cardiology* 14(3), 198–205. 10.1177/1474515114522563 24491348

[CIT0035] Taylor N.F., Dodd K.J., Shields N. & Bruder A., 2007, ‘Therapeutic exercise in physiotherapy practice is beneficial: A summary of systematic reviews 2002-2005’, *The Australian Journal of Physiotherapy* 53(1), 7–16, viewed 11 July 2011, from http://www.ncbi.nlm.nih.gov/pubmed/17326734. 17326734 10.1016/s0004-9514(07)70057-0

[CIT0036] Tofthagen C., Visovsky C. & Berry D.L., 2012, ‘Strength and balance training for adults with peripheral neuropathy and high risk of fall: Current evidence and implications for future research’, *Oncology Nursing Forum* 39(5), E416–E424. 10.1188/12.ONF.E416-E424 22940521 PMC5385995

[CIT0037] Tumusiime D.K., Venter F., Musenge E. & Stewart A., 2014a, ‘Prevalence of peripheral neuropathy and its associated demographic and health status characteristics, among people on antiretroviral therapy in Rwanda’, *BMC Public Health* 14, 1306 10.1186/1471-2458-14-1306 25526665 PMC4320525

[CIT0038] Tumusiime D.K., Stewart A., Venter F.W.D. & Musenge E., 2014b, ‘The reliability of the modified lower extremity functional scale among adults living with HIV on antiretroviral therapy, in Rwanda, Africa’, *Sahara Journal* 11(1), 178–186. 10.1080/17290376.2014.976249 25383643 PMC4272140

[CIT0039] Wang S.X., Ho E.L., Grill M., Lee E., Peterson J., Robertson K. et al., 2014, ‘Peripheral neuropathy in primary HIV infection associates with systemic and CNS immune activation’, *Journal of Acquired Immune Deficiency Syndromes (1999)* 66(3), 303–310. 10.1097/QAI.0000000000000167 24732871 PMC4147038

[CIT0040] White C.M., Pritchard J. & Turner-Stokes L., 2004, ‘Exercise for people with peripheral neuropathy’, *The Cochrane Database of Systematic Reviews* 4, CD003904 10.1002/14651858.CD003904.pub2 PMC12767756 15495069

[CIT0041] Yaksh T.L. & Sorkin L.S., 2005, ‘Mechanisms of neuropathic pain’, *Current Medicinal Chemistry – Central Nervous System Agents* 5(2), 129–140. 10.2174/1568015054022380

[CIT0042] Zakas A., Grammatikopoulou M.G., Zakas N., Zahariadis P. & Vamvakoudis E., 2006, ‘The effect of active warm-up and stretching on the flexibility of adolescent soccer players’, *The Journal of Sports Medicine and Physical Fitness* 46(1), 57–61, viewed 19 October 2012, from http://www.ncbi.nlm.nih.gov/pubmed/16596100. 16596100

